# Assessing the genetic burden of familial hypercholesterolemia in a large middle eastern biobank

**DOI:** 10.1186/s12967-022-03697-w

**Published:** 2022-11-03

**Authors:** Geethanjali Devadoss Gandhi, Waleed Aamer, Navaneethakrishnan Krishnamoorthy, Najeeb Syed, Elbay Aliyev, Aljazi Al-Maraghi, Muhammad Kohailan, Jamil Alenbawi, Mohammed Elanbari, Qatar Genome Program Research Consortium (QGPRC), Borbala Mifsud, Younes Mokrab, Charbel Abi Khalil, Khalid A. Fakhro

**Affiliations:** 1 College of Health and Life Sciences, Hamad Bin Khalifa University (HBKU), Doha, Qatar; 2 Human Genetics Department, Sidra Medicine, Doha, Qatar; 3 Bioinformatics, Genomic Data Science Core, Sidra Medicine, Doha, Qatar; 4 Clinical Research Centre, Sidra Medicine, Doha, Qatar; 5 Laboratory of Medical and Population Genomics, Sidra Medicine, Doha, Qatar; 6 Department of Genetic Medicine, Weill Cornell Medicine, Education City, Qatar; 7 Joan and Sanford I. Weill Department of Medicine, Weill Cornell Medicine, New York, US

**Keywords:** Cholesterol, Dyslipidemias, LDL, Lipoproteins/Receptors, Premature coronary artery disease, Dutch lipid Clinic Network, *LDLRAP1*, Sitosterolemia, Polygenic risk scores, Middle East region.

## Abstract

**Background:**

The genetic architecture underlying Familial Hypercholesterolemia (FH) in Middle Eastern Arabs is yet to be fully described, and approaches to assess this from population-wide biobanks are important for public health planning and personalized medicine.

**Methods:**

We evaluate the pilot phase cohort (n = 6,140 adults) of the Qatar Biobank (QBB) for FH using the Dutch Lipid Clinic Network (DLCN) criteria, followed by an in-depth characterization of all genetic alleles in known dominant (*LDLR*, *APOB*, and *PCSK9*) and recessive (*LDLRAP1*, *ABCG5*, *ABCG8*, and *LIPA*) FH-causing genes derived from whole-genome sequencing (WGS). We also investigate the utility of a globally established 12-SNP polygenic risk score to predict FH individuals in this cohort with Arab ancestry.

**Results:**

Using DLCN criteria, we identify eight (0.1%) ‘definite’, 41 (0.7%) ‘probable’ and 334 (5.4%) ‘possible’ FH individuals, estimating a prevalence of ‘definite or probable’ FH in the Qatari cohort of ~ 1:125. We identify ten previously known pathogenic single-nucleotide variants (SNVs) and 14 putatively novel SNVs, as well as one novel copy number variant in *PCSK9*. Further, despite the modest sample size, we identify one homozygote for a known pathogenic variant (*ABCG8*, p. Gly574Arg, global MAF = 4.49E-05) associated with Sitosterolemia 2. Finally, calculation of polygenic risk scores found that individuals with ‘definite or probable’ FH have a significantly higher LDL-C SNP score than ‘unlikely’ individuals (p = 0.0003), demonstrating its utility in Arab populations.

**Conclusion:**

We design and implement a standardized approach to phenotyping a population biobank for FH risk followed by systematically identifying known variants and assessing putative novel variants contributing to FH burden in Qatar. Our results motivate similar studies in population-level biobanks – especially those with globally under-represented ancestries – and highlight the importance of genetic screening programs for early detection and management of individuals with high FH risk in health systems.

**Supplementary Information:**

The online version contains supplementary material available at 10.1186/s12967-022-03697-w.

## Background

Familial hypercholesterolemia (FH) is an autosomal-dominant genetic disorder characterized by elevated plasma low-density lipoprotein cholesterol (LDL-C) levels, with a prevalence between 1:250 and 1:500 across different world populations [1–4]. When left untreated, FH increases the risk of premature coronary artery disease (CAD), with an estimated 20% of myocardial infarctions (MIs) in patients aged under 45 years attributable to FH [2]. FH should be suspected in adults with LDL-C > 4.9 mmol/L (190 mg/dL) and children with levels > 4 mmol/L (160 mg/dL) combined with a family history of premature CAD [5–7]. There are three formal diagnostic criteria widely used to diagnose FH: the Dutch Lipid Clinic Network (DLCN) [8, 9], Simon Broome [10], and Make Early Diagnosis to Prevent Early Death (MEDPED) criteria [11]. Of these three sets, the DLCN and Simon Broome criteria rely on genetic variations present in FH causing genes combined with other clinical features.

To date, pathogenic variants causing FH are predominantly reported in three genes: low-density lipoprotein receptor (*LDLR*), 95%; apolipoprotein B (*APOB*), 2–11%; and proprotein convertase subtilisin/kexin type 9 (*PCSK9*), 1% [1, 8, 9, 12]. Also, some recessive genes have been associated with FH, including Low-Density Lipoprotein Receptor Adaptor Protein 1 *(LDLRAP1)*, ATP Binding Cassette Subfamily G Member 5 (*ABCG5)*, ATP Binding Cassette Subfamily G Member 8 (*ABCG8)*, and Lipase A, Lysosomal Acid Type (*LIPA)*.

Polygenic inheritance is the most likely cause of disease in patients with a clinical diagnosis of FH without detectable variants in the *LDLR*, *APOB*, and *PCSK9* genes (variants in the novel genes were observed only in few cases) [13]. In 2013, Talmud et al. developed a 12-SNP LDL-C “SNP-Score” based on common variants identified in genome wide association studies that were associated with increased LDL-C levels [13, 14]. Validation of this score in European-Caucasian population has shown that 80% of the clinically diagnosed FH patients with no detectable mutations in *LDLR, APOB*,and *PCSK9* have a polygenic inheritance [13].

Although FH is primarily caused by dominant variants; rare cases have been found to harbor homozygous variants (prevalence 1:160,000–1:300,000) [9]. The incidence of homozygous FH (HoFH) is increased in Middle Eastern countries due to the high degree of consanguinity. For example, the homozygous *LDLR* allele (p.C681X) is responsible for 60% of FH cases in Lebanon [15]. There is another form of HoFH caused by biallelic variants in the *LDLRAP1* gene, termed autosomal recessive hypercholesterolemia (ARH). ARH was first described by Khachadurian and Uthman in Lebanese families in 1973 [16] with a global prevalence of less than 1 in 1 million [17]. However, ARH is found more commonly on Sardinia Island in Italy due to founder effect and inbreeding. About 100 ARH patients have been reported so far, most of them from Sardinian Island [18]. The prevalence of ARH in Sardinian Island was estimated to be 1 in 40,000, and the frequency of heterozygous carriers is 1:143 [17]. ARH is also characterized by a severe elevation in the LDL-C levels, tendon xanthomas, and premature CAD [19]. Half of the ARH patients reported have evidence of CAD [18]; however, no ARH patients with premature CAD have been reported before 20 years old [20].

A recent census of FH cases in the Arabian Gulf (Kuwait, Oman, Qatar, Saudi Arabia, and the United Arab Emirates) showed 130,693 heterozygous carriers and 87 HoFH cases [21]. Notably, the EAS Familial Hypercholesterolaemia Studies Collaboration (FHSC) reported 57 FH genetic variants in 17 Middle Eastern and North African countries, while none were identified in Qatar [21]. Similarly, Alhababi and Zayed (2018) reported that no FH-related genetic variants had been found in 14 Arab countries, including Qatar [22]. Thus, the identity and prevalence of FH variants in the Qatari population have not been well established.

In the present study, a large Arab population biobank has been utilized to assess the genetic burden of FH in a systematic and large-scale manner, which may serve as a reference dataset for future studies of FH in the region. We conducted the large-scale characterization of FH alleles in any Arab population, using a whole-genome sequencing (WGS) dataset of 6,140 adult participants from Qatar Genome Program (QGP). We used the extensive phenotypic data from Qatar BioBank (QBB) for the FH diagnosis of 6,140 participants using DLCN criteria. We assessed the presence of known pathogenic variants in *LDLR*, *APOB*, *PCSK9, LDLRAP1*, *ABCG5*, *ABCG8*, and *LIPA* in these individuals and evaluated novel variants of these genes for pathogenicity. Furthermore, we tested the utility of globally established 12 SNP LDL-C SNP scores for predicting polygenic FH risk in Arab populations.

## Methods

### Cohort description

The study participants were recruited by Qatar Biobank (QBB), a prospective, population-based cohort established in 2012 involving the study of adult Qatari nationals and long-term residents (≥ 15 y of continuous residence) who were followed up every five years [23, 24]. Initial analysis was started with 6,218 participants; however, 78 participants were excluded due to the lack of LDL-C levels leaving 6,140 participants for this study. Among 6,140 participants, no one reported or appeared to have hypothyroidism. The whole-genome sequence (WGS) of these participants was sequenced through Qatar Genome Program (QGP).

### Lipid measurement and correction factor for cholesterol-lowering medications

Blood samples were collected from QBB participants and stored at − 80 °C [23, 24]. Enzymatic calorimetric assays were performed to analyze the lipid profiles (total cholesterol, LDL cholesterol, HDL cholesterol and triglycerides) using a Roche Cobas analyzer at Hamad Medical Corporation Laboratory, Doha, Qatar. When a QBB participant reported using cholesterol-lowering medication, LDL-C concentrations were corrected to estimate pre-treated LDL-C levels as described previously (Supplementary Table 1) [25]. For the participants under treatment with unspecified cholesterol-lowering medication, a correction factor of 1.43 was used, which corresponded to an estimated 30% reduction in LDL-cholesterol [26].

### Co-morbidities

In the present study, coronary artery disease (CAD) included angina pectoris and myocardial infarction. QBB questionnaires (nurse interview) were used to identify participants diagnosed with these conditions (self-reported). Similarly, cholesterol-lowering medications, diabetes mellitus, hypertension and parents’ history of coronary artery diseases were self-reported on the QBB questionnaire. ‘Smokers’ indicated current smokers. Body mass index (BMI) was calculated as weight (kg) divided by height squared (m2), and body composition was ascertained using a SECA 514 mBCA. The metabolic syndrome was defined in accordance with international guidelines [27].

### Diagnostic criteria for FH

The DLCN criteria as modified and used in our study included family history of a first-degree relative with CAD or vascular disease, a personal history of premature CAD, or premature cerebral or peripheral vascular disease, and elevated LDL-C levels. Each of these criteria was given a score and FH diagnosis was classified as follows: a score of 8 and above - ‘definite’ FH; a score between 6 and 8 - ‘probable’ FH; a score between 3 and 5 - ‘possible’ FH; and score of less than 3 were classified as ‘unlikely’ FH [8, 9].

### Whole genome sequencing

The DNA extraction, library construction and whole genome sequencing (WGS) of QBB samples have been performed at Sidra Medicine; full details of the WGS data have been published previously [28–30]. An automated pipeline has been developed by the Sidra Bioinformatics Core for performing standardized quality control and variant calling on whole genome sequence data; details of the data have been previously described [28–30]. Genetic architecture of the Qatari population in relation to the world’s population reveals five major ancestries, namely General Arabs (QGP_GAR), Peninsular Arabs (QGP_PAR), Arabs of Western Eurasia and Persia (QGP_WEP), South Asians (QGP_SAS), Africans (QGP_AFR) and Admixed (QGP_ADM). A full description of the genetic architecture study is provided in Razali et al. (2021)[30]. According to this study, the PAR cluster is unique to Qataris. GAR cluster overlap with the Levant (including both Arab and Jewish populations) and North Africa, while WEP clusters mainly overlap with Persians, Turkish and other West Eurasian groups. Finally, AFR and SAS sub-clusters exhibit similarities to other Eastern African and South Asian populations, respectively.

### Estimated clinical penetrance and ACMG (the American College of Medical Genetics and Genomics) classification

Estimated clinical penetrance for ClinVar, HGMD and novel variants were calculated based on the total number of definite, probable, and possible FH individuals of DLCN criteria carrying the variants [31]. ACMG classification of novel variants was obtained using online bioinformatic software, InterVar [32].

#### Copy number variant analysis

Structural variants (SVs) in the WGS data were called using two structural variant callers: Manta version 1.6.0 and SpeedSeq version 0.1.2 using default parameters. Size cut-offs were set at ≥50 bp and ≤10 Mb. We generated a consensus file for each individual by merging SVs that overlap reciprocally by > 80%. A consensus multi-sample SV dataset was created and annotated using AnnotSV 2.2. In this study, we focused on SVs disrupting *LDLR*, *PCSK9* and *APOB*.

#### Structural mapping of novel variants

A structural analysis was performed on the novel variants of *LDLR* and *PCSK9*. Literature-based study and the X-ray crystallography 3D structures of PCSK9 protein (PDB code 2P4E, 6U2F, 5VLP, 2PMW, 6U2F, 3BPS, 6U2N, 6U36, 6U3I, and 6U26) were used to map the functional domains, novel and known mutational positions, allosteric inhibitor site residues, LDLR binding surface, catalytic triad, substrate-binding pocket and the interface used for the interaction of the pro-domain with the catalytic domain. To analyze the protein interaction of LDLR with PCSK9, we used the following complex 3D structures: 1N7D, 3M0C, 3P5C, 1IJQ, and 3P5B. Schematic representations of the structures were generated using PYMOL (The PyMol Molecular Graphics System, Schrodinger, LLC).

### Polygenic risk score calculation

To study the possible polygenic cause of hypercholesterolemia, we focused on a compilation of 12 key single nucleotide polymorphisms (SNPs) that significantly raise the LDL-C [14, 33–35]. Since the one of the 12 SNP rs1800562 in the *HFE* gene was not found in the QGP data, our LDL-C SNP score calculation was based on 11 of the 12 key SNPs. LDL-C SNP scores were calculated as the weighted sum of the LDL-C-raising alleles, where weights are the effect sizes in genome-wide association studies (GWAS) (Supplementary Table 2). The summary statistics of the 11 SNPs were obtained from the Global Lipid Genetics Consortium GWAS [13, 36].

### Statistical analysis

All statistical analysis in this study were performed using R (version 1.1.453). We evaluated the prevalence of cardiovascular risks and other co-morbidities in ‘definite or probable’ FH individuals versus ‘unlikely’ FH individuals using the Chi-square test (significance at p < 0.01). We compared the SNP LDL-C gene score between ‘definite or probable FH’, ‘possible FH’, and ‘unlikely’ FH groups using one-way ANOVA. The odds ratios (ORs) for having LDLR variants in ‘definite or probable’ FH, and ‘possible’ FH were compared with those for ‘unlikely’ FH.

## Results

### Demographic and clinical characteristics of Qatar Biobank participants

**Fig. 1:**
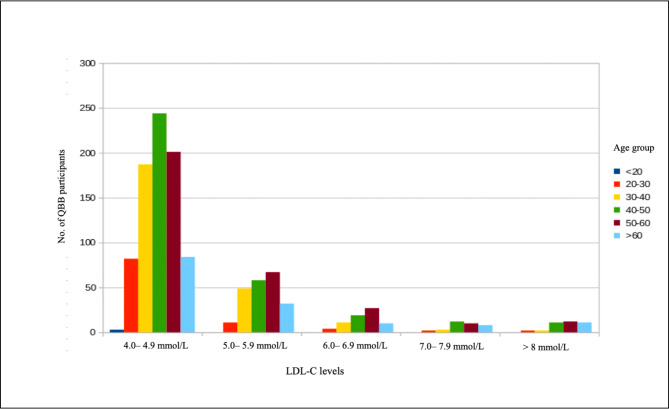
Distribution of higher LDL-C levels (≥ 4 mmol/L) among QBB participants according to their age group

A total of 6,140 participants were included in this study. The median participant age was 39 y (interquartile range:18–88), and 57% were female (Table 1). A total of 1,854 (30.1%) participants had a self-reported history of hypercholesterolemia (Table 1); 400 had LDL-cholesterol levels ≥ 4.9 mmol/L (Table 1; Fig. 1). We observed 586 individuals who reported taking a cholesterol-lowering medication and another 237 individuals who reported taking cholesterol-lowering medications along with diet management. Notably, 36 participants reported having high cholesterol before or at the age of 20. Further, 57 participants were diagnosed with premature CAD (31 with myocardial infarction and 26 with angina).

**Table 1:** Qatar Biobank (QBB) data associated with hypercholesterolemia phenotype

QBB phenotypic data	No. of QBB participants
Number of participants	6140
Gender (Male, Female)	2645, 3495
Parental consanguinity	2347
Age (median)	39
**LDL-C**:	
< 4.9mmol/L	5740
≥ 4.9mmol/L	400
Self-reported high cholesterol	1854
No. of participants reported having high cholesterol ≤ 20 years old	36
**Self-reported treatment for high cholesterol**:	
Diet only	413
Diet and Tablets	237
Tablets only	586
**Self-reported cardiovascular events**	
Heart attack	31
Angina	26
Stroke	10
Revascularization surgery	69
**First-degree relatives with history of myocardial infarction**	
Parent history of myocardial infarction	862
Siblings’ history of myocardial infarction	4

LDL-C levels shown are corrected or pre-treated LDL-C levels obtained using specific correction factors (see methods)

### FH diagnosis

We sought to classify the FH status of all QBB participants using the Dutch Lipid Criteria Network (DLCN) criteria (see Methods). We identified eight (0.1%) ‘definite’, 41 (0.7%) ‘probable’ and 334 (5.4%) ‘possible’ FH individuals; the remaining 5,757 individuals were classified as ‘unlikely FH’ (Table 2). Thus, we estimated a prevalence of 0.8% (1:125) for ‘definite or probable’ FH within the QBB cohort.

**Table 2:** Dutch Lipid Clinic Network criteria as modified and used in this study

Dutch Lipid Clinic Network (DLCN)	Points	No. of QBB participants (n=6140)
**Family History**		
First-degree relative with known coronary and vascular disease	1	862
**Clinical history**		
Patient with premature* coronary artery disease	2	50
Patient with premature* cerebral or peripheral vascular disease	1	6
**LDL-C (mmol/liter)**		
LDL-C (≥ 8.5)	8	28
LDL-C (6.5–8.4)	5	75
LDL-C (5.0–6.4)	3	264
LDL-C (4.0–4.9)	1	801
**Diagnosis**		
Definite FH	> 8	8
Probable FH	6 – 8	41
Possible FH	3 – 5	334
Unlikely FH	<3	5757

*Premature: ≥55 years for men; ≥60 years for women

### Cardiovascular risks and other co-morbidities

Our study found that 8% of participants classified as ‘definite or probable’ FH had self-reported premature CAD, which is significantly higher than the ‘unlikely’ FH individuals (0.4%) (*χ*^2^*P < 0.01)* (Table 3). Furthermore, 6% of ‘definite or probable’ FH individuals underwent heart revascularization surgery as compared to 1% in ‘unlikely’ FH (*χ*^2^*P < 0.01)*. Additionally, we found that the presence of co-morbidities such as metabolic syndrome, hypertension, and diabetes mellitus was significantly higher among participants classified as ‘definite or probable’ FH, compared to unlikely FH (metabolic syndrome 47%, hypertension 31% and diabetes mellitus 47% in ‘definite or probable’ FH vs 15% metabolic syndrome, 15% hypertension, and 16% diabetes mellitus in ‘unlikely’ FH) (*χ*^2^*P < 0.01)*. Finally, when including the parental history taken in at the time of biobank enrollment, we found that 100% definite, 44% probable and 22% possible FH individuals reported either their mother or father had died from a myocardial infarction compared to 13% in unlikely FH.

**Table 3:** Co-morbidities of 6,140 Qatar Biobank (QBB) participants categorized by the Dutch Lipid Clinic Network criteria

Criteria	Dutch Lipid Clinic Network criteria			
	Definite	Probable	Possible	Unlikely
**Prevalence**	8	41	334	5757
**Sex, women**	4	22	159	3310
**Age, years**	59 (36–69)	54 (20–70)	49 (21–80)	38 (18–88)
**Body mass index**	30.48 (25.9–36.9)	28.8 (22.6–40.7)	29.6 (17.5–53.3)	28.7 (13.4–110.4)
**Obesity**	6	8	172	2568
**Metabolic syndrome**	4	19	122	870
**Diabetes mellitus**	3	20	112	912
**Hypertension**	2	13	117	855
**Current smokers**	3	3	56	895
**Cholesterol-lowering medication**	7	34	218	623
**Premature coronary artery disease**	0	4	21	25
**Myocardial infarction**	0	2	12	11

Age and body mass index (BMI) are summarized as median and interquartile range. Body mass index was measured in kg/m^2^. Obesity was defined as “body mass index ≥ 30kg/m^2^”. The metabolic syndrome was defined in accordance with international guidelines (Alberti et al. 2013). Details of diabetes mellitus, hypertension, and smoking history were self-reported and obtained from the QBB questionnaire

### The genetic spectrum of known pathogenic FH variants in LDLR, APOB and PCSK9

To identify known pathogenic variants associated with FH segregating in the Qatari population, we annotated the genomic data against the ClinVar and Human Genome Mutation Database (HGMD). We found four SNVs previously reported as pathogenic/likely pathogenic (P/LP) in ClinVar were present in 11 heterozygous individuals (0.18%) (Table 4). As noted, all four variants were in *LDLR*, and one was a loss-of-function (LoF) variant (c.313 + 3 A > C) that was found in six heterozygous individuals [37], while the remaining three were missense variants (Table 4). We found two *APOB* missense variants associated with hypobetalipoproteinemia and each variant was identified in two heterozygous individuals. Heterozygous individuals (n = 4) carrying the two *APOB* variants have LDL-C levels below the 5th percentile (LDL-C < 1.8 mmol/L) of the general population (Table 4). Their APOB protein levels, however, were not able to be assessed since QBB does not have this information.

**Table 4:** ClinVar pathogenic/likely pathogenic variants of familial hypercholesterolemia in the Qatar Genome Program study

Gene	dbSNP	cDNA change	Amino-acid change	QGP AC	Max AF in public databases	QGP subcluster	Estimated clinical penetrance	ClinVar	ClinVar Phenotype
***ABCG5***	rs199689137	c.1336 C>T	p.Arg446*	27	0.0006	QGP_ADM (1), QGP_GAR(24), QGP_PAR(1), QGP_WEP (1)	.	P	Sitosterolemia 1
***ABCG8***	rs137852991	c.1234 C>T	p.Arg412*	1	6.67E-05	QGP_ADM	.	P	Sitosterolemia 2
***ABCG8***	rs137852988	c.1720G>A	p.Gly574Arg	6	4.49E-05	QGP_WEP	100% (1/1)*	P/LP	Sitosterolemia 2
***APOB***	.	c.2817-2 A>C	.	2	0	QGP_ADM	.	LP	Hypobetalipoproteinemia
***APOB***	.	c.1468 C>T	p.Arg490Trp	2	1.79E-05	QGP_ADM (1), QGP_GAR (1)	.	P	Hypobetalipoproteinemia
***LDLR***	rs879254809	c.1154T>G	p.Leu385Arg	1	0	QGP_AFR	0% (0/1)	LP	Familial Hypercholesterolemia
***LDLR***	rs758194385	c.1691 A>G	p.Asn564Ser	1	1.74E-04	QGP_GAR	100% (1/1)	LP	Familial Hypercholesterolemia
***LDLR***	rs771019366	c.269 A>G	p.Asp90Gly	3	8.24E-06	QGP_WEP(2), QGP_ADM(1)	67% (2/3)	P/LP	Familial Hypercholesterolemia
***LDLR***	rs1064793799	c.313+3 A>C	.	6	0	QGP_PAR(6)	83% (5/6)	P	Familial Hypercholesterolemia
***LIPA***	.	c.863 C>T	p.Thr288Ile	1	0	QGP_SAS	.	LP	Lysosomal acid lipase deficiency

QGP_AC: QGP allele count. The maximum AF reported was observed in ExAC and gnomAD exome databases. The numbers in brackets in QGP_subclusters represent the allele count belonging to a specific sub-cluster carrying the variant. General Arabs (QGP_GAR), Peninsular Arabs (QGP_PAR), Arabs of Western Eurasia and Persia (QGP_WEP), South Asians (QGP_SAS), Africans (QGP_AFR) and Admixed (QGP_ADM). Estimated clinical penetrance was calculated based on the total number of definite, probable, and possible FH individuals carrying the variants in the QBB cohort. * Estimated clinical penetrance was calculated for the homozygous individual carrying the *ABCG8* recessive variant. Clinvar significance: P – Pathogenic; LP – Likely Pathogenic

Using HGMD classification, we identified 28 variants (22 in *LDLR*, 4 in *PCSK9*, and 2 in *APOB*) reported as disease-causing mutations (DM) (Supplementary Table 3). Analyzing the estimated clinical penetrance of DM variants, we found that four *LDLR* variants showed 100% penetrance, each with one affected individual. A further six DM variants showed incomplete penetrance, and the remaining eighteen DM variants showed zero penetrance, suggesting that the HGMD variant annotation is less specific than the ClinVar annotation (Supplementary Table 3).

### Identification of candidate novel pathogenic FH variants in LDLR, PCSK9 and APOB

**Fig. 2:**
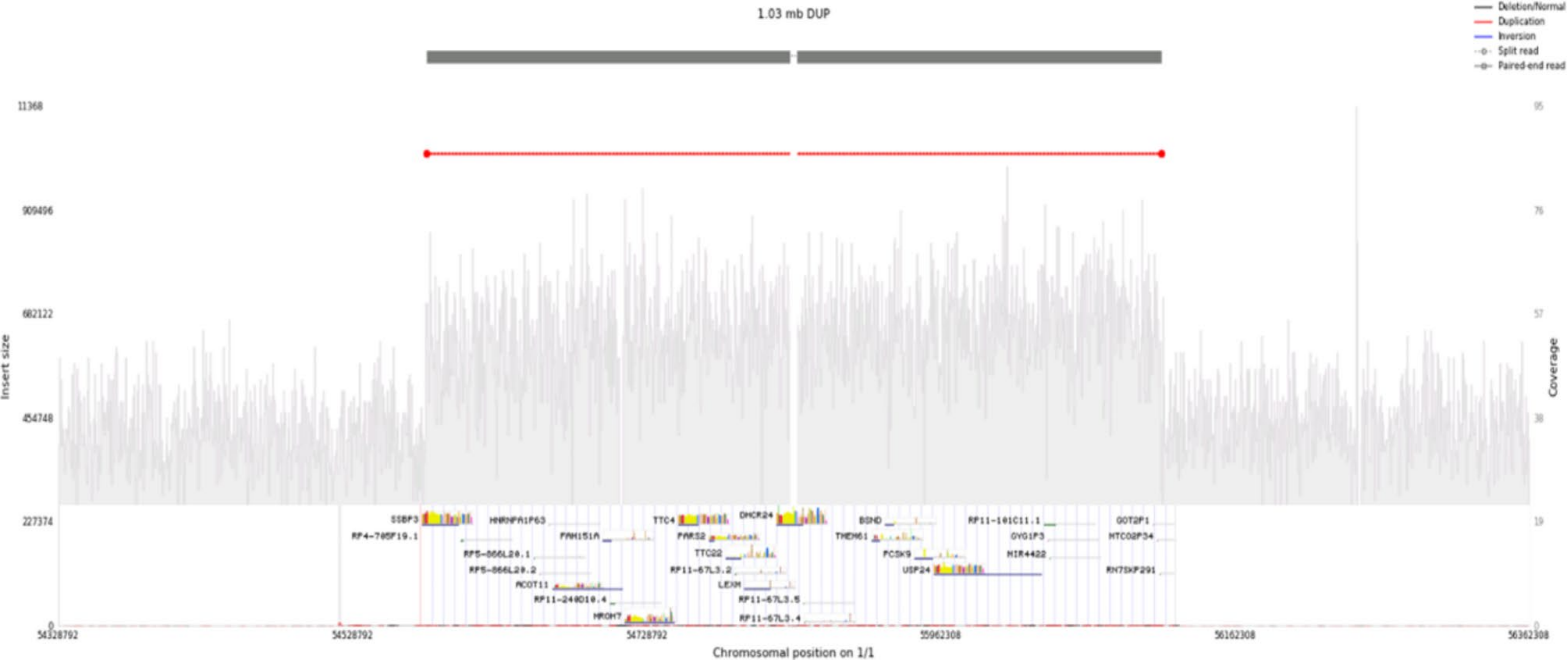
Structural variant analysis of loci 1:54828792-55862308 showing gene duplication in *PCSK9* gene. Next-generation sequencing (NGS)-based detection of a *PCSK9* copy number variation (CNV) in a likely FH individual. The duplication is marked by an increase in average read depth within the interval of (Chr1:54,828,792-55,862,308, 1.03 Mb) and is supported by paired-end reads (red boxes connected by thin line) that map to either side of the affected allele, confirming the duplication breakpoints. Region affected by duplication covers all 12 exons of the *PCSK9* gene, plus the rs11206510 probe 8,655 bases upstream of *PCSK9.*

In addition to known pathogenic alleles, we sought to identify putatively novel deleterious variants disrupting the three canonical FH genes. Leveraging our pipeline to call both small and large genomic variants from the WGS data, we identified one individual with a 1.03 Mb duplication (chr1:54,828,792 − 55,862,308) encompassing *PCSK9* (Fig. 2). As expected with excess *PCSK9* dosage, the individual had a high LDL-C level of 6.03 mmol/L (> 97th percentile).

We identified a further 11 novel putatively deleterious SNVs (missense or LoF, minor allele frequency (MAF) < 0.1% in QGP) in the three genes (7 in *APOB*, 3 in *PCSK9* and 1 in *LDLR*). While each novel variant was observed in at least one ‘definite or probable or possible’ FH individual with a history of self-reported hypercholesterolemia and LDL-C ≥ 4.9 mmol/L, overall, with estimated clinical penetrance ranging from 14 to 100% (Table 5). ACMG classification of these novel variants revealed that only two were ‘likely pathogenic’ (*PCSK9*, p. Gly59Arg, and *LDLR* p. Asp472Asn), with the remaining classified as VUS. Functional and molecular characterization of novel variants is required to support their pathogenicity further.

**Table 5:** Fourteen novel putative pathogenic variants associated with familial hypercholesterolemia in the Qatar Genome Program study

Gene	HGV_DNA	HGV_P	QGP AC	QGP subclusters	DLCN criteria	Estimated clinical Penetrance	LDL-C (mmol/L)	Self-reported HC	ACMG classification
*ABCG8*	c.391 C>T	p.Gln131*	2	QGP_WEP	.	.	.	.	**LP** (PVS1, PM2, PP3)
*APOB*	c.8936G>A	p. Gly2979Asp	1	QGP_ADM	1 Probable	100% (1/1)	8.7	Yes	**VUS** (PM2, BP4)
*APOB*	c.7106 A>G	p. Lys2369Arg	2	QGP_WEP	1 Probable	50% (1/2)	7.7	Yes	**VUS** (PM2, BP4)
*APOB*	c.4412T>G	p. Leu1471Trp	5	QGP_ADM (1), QGP_WEP (4)	1 Possible	20% (1/5)	6	Yes	**VUS** (PM2, BP4)
*APOB*	c.9336G>T	p. Glu3112Asp	3	QGP_WEP	1 Probable	33% (1/3)	9.2	Yes	**VUS** (PM2)
*APOB*	c.9547 A>G	p. Arg3183Gly	6	QGP_GAR	1 Possible	16% (1/6)	6.1	Yes	**VUS** (PM2, BP4)
*APOB*	c.1697T>C	p. Met566Thr	11	QGP_GAR (4), QGP_PAR (7)	2 Possible	18% (2/11)	5.6, 6.6	Yes, yes	**VUS** (PM2)
*APOB*	c.4780 C>A	p. Gln1594Lys	10	QGP_WEP	1 Probable, 1 Possible	20% (2/10)	6.6, 6	Yes, yes	**VUS** (PM2)
*LDLR*	c.1414G>A	p. Asp472Asn	8	QGP_ADM (1), QGP_GAR (7)	1 Possible	14% (1/8)	5.1	Yes	**LP** (PM1, PM2, PP2, PP3, BP1)
*LDLRAP1*	C.200 C>T	p. Ser67Leu	39	QGP_WEP	.	0% (0/1) *	.	Yes	**VUS** (PM1, PM2, PP4)
*LIPA*	C.149 A>C	p.Glu50Ala	1	QGP_ADM	.	.			**LP** (PM1, PM2, PM5, PP3)
*PCSK9*	c.175G>C	#p. Gly59Arg	1	QGP_AFR	1 Possible	100% (1/1)	6	Yes	**VUS** (PM2, BP4)
*PCSK9*	c.203 C>A	#p. Ala68Asp	1	QGP_AFR	1 Possible	100% (1/1)	6	Yes	**VUS** (PM2)
*PCSK9*	c.908G>A	p. Arg303His	3	QGP_ADM (2), QGP_WEP (1)	1 Possible	33% (1/3)	6.5	Yes	**VUS** (PM1, PM2)

Self-reported HC- Self-reported hypercholesterolemia. # Both these *PCSK9* variants are present in the same individual. * Estimated clinical penetrance was reported for the homozygous individual carrying the *LDLRAP*1 variant. LDL-C levels and history of self-reported hypercholesterolemia were reported for individuals classified as ‘definite or probable or possible’ FH carrying the novel variants. InterVar (automated ACMG classifier) was used for novel variants classification based on ACMG/AMP 2015 guidelines. VUS - Variants of uncertain significance and LP - likely pathogenic

### In silico characterization of novel variants

**Fig. 3:**
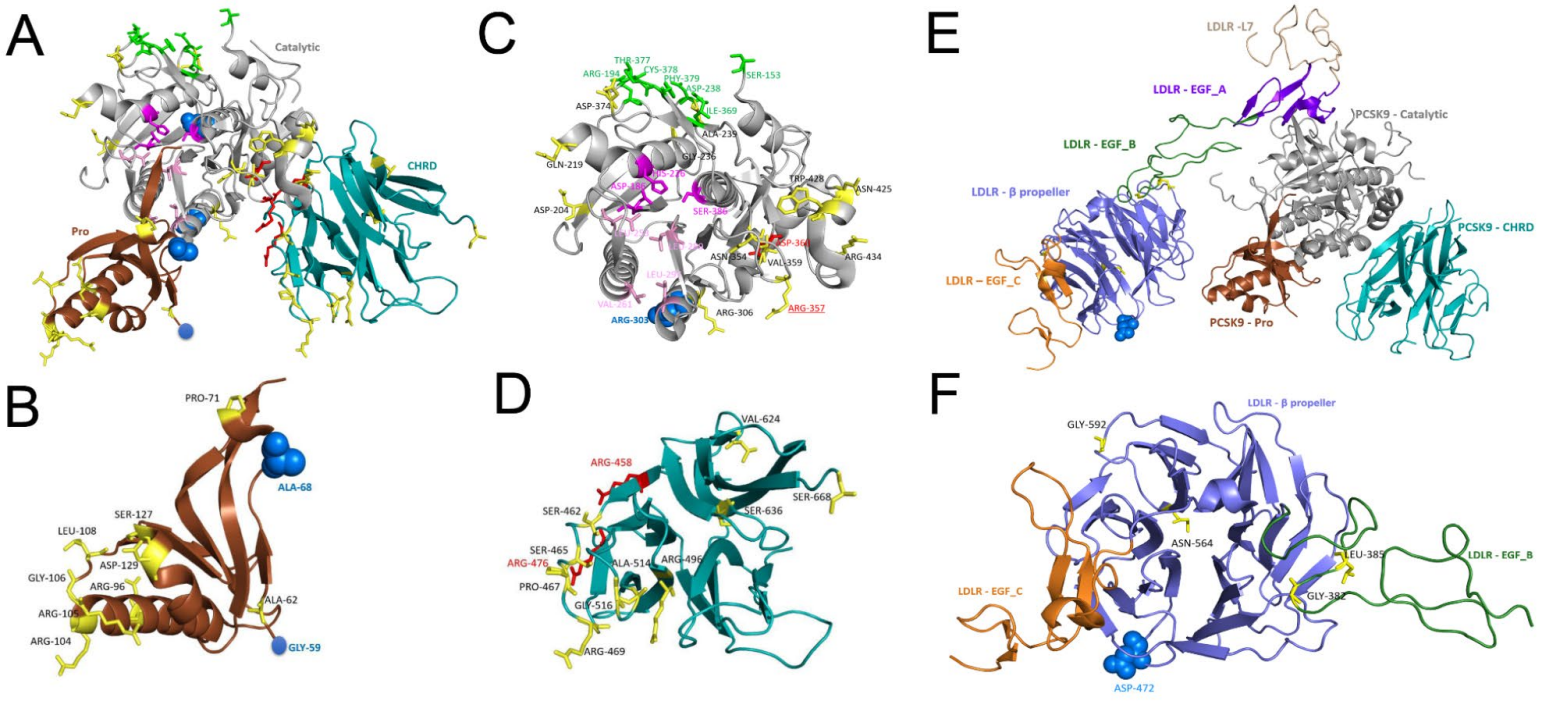
Mapping of key regions in the 3D structure of PCSK9 and LDLR. A) Structure of PCSK9 showing arrangement of functional domains, pro-domain (brown), catalytic domain (gray) and cystine and histidine-rich domain (CHRD, cyan). (B-D) Zoomed in view of the domains [(B) pro-domain, (C) catalytic domain (D) CHRD (also binding region for annexin A2)] with labels. E) Structure of a PCSK9-LDLR complex shown with an epidermal growth factor (EGF) domain composed of EGF_A (purple), EGF_B (forest), EGF_C (orange) and β-propeller regions (slate). F) Zoomed in view of the β-propeller with neighbor domains. In this figure, positions are mapped and colored as follows: novel mutations, blue spheres; known mutations, yellow; catalytic triad, magenta; substrate binding region, pink; LDLR (EGF_A) binding region in PCSK9, green; allosteric inhibitor site, red

We sought to characterize the effect of candidate novel *PCSK9* and *LDLR* variants based on *in silico* modelling of 3D protein structure. The PCSK9 protein comprises three domains: a pro-domain, which controls folding and acts as an inhibitor of catalytic activity [38, 39] ; a catalytic domain, which regulates protease activity and interacts with the LDLR [40]; and a cystine histidine-rich domain (CHRD), which binds annexin A2 to suppress LDL-C levels [41, 42]. By examining the three novel variants of PCSK9 two of them (Gly59Arg, Ala68Asp) occurring in the pro-domain (Fig. 3A-B) in the same individual, a 25- year-old male classified as ‘possible’ FH, with LDL-C levels of 6 mmol/L and self-reported hypercholesterolemia. Examination of the WGS read data confirmed these two variants were on the same haplotype (Supplementary Fig. 1), but only the (Gly59Arg) variant was scored as pathogenic by the InterVar ACMG/AMP 2015 guidelines. The third variant (Arg303His), which affects the catalytic domain (Fig. 3A and C), was shared by three individuals. However, only one was classified as ‘possible’ FH (a 25-year-old male with an LDL-C levels of 6.5 mmol/L and self-reported history of hypercholesterolemia). The other two carriers were 27 and 43 years old, with LDL-C levels of 2.5 mmol/L and 3.8 mmol/L, respectively, suggesting incomplete penetrance.

To map our novel *LDLR* variant (p. Asp472Asn), we studied PCSK9-LDLR complex structures. In mammalian cells, PCSK9 binds to LDLR and regulates cholesterol levels [43]. This process requires interactions between several structural domains of these proteins (Fig. 3E). The LDLR protein contains a ligand-binding domain (L1–L7 repeats; approximately 40 residues each), and an epidermal growth factor precursor homology domain (EGFPH) composed of EGF_A, EGF_B, β-propeller (six-bladed) and EGF_C regions [44, 45]. Asp472Asn maps to the β-propeller of the LDLR (Fig. 3E and F), a region involved in the regulation of the open (active) and closed (inactive) states of the PCSK9-LDLR complex. This suggest that the variant may play a role in the binding of LDLR with the pro-domain of PCSK9 in a process that is key to LDLR recycling. We were unable to assess the novel genetic variation in *APOB* since the protein crystal structure is unavailable.

### Known and novel putative pathogenic variants in recessive FH genes

We assessed the known and novel putative pathogenic variants in four recessive FH genes *LDLRAP1, ABCG5, ABCG8*, and *LIPA*. We found one disease-causing (DM) variant (p. Ser202Tyr) (Supplementary Table 3) and one predicted pathogenic novel variant (p. Ser67Leu), but no ClinVar reported P/LP variants in *LDLRAP1* gene associated with autosomal recessive hypercholesterolemia (ARH) (Table 5). The first variant (p. Ser202Tyr) is present in 132 heterozygous carriers and three homozygous individuals (QGP MAF 1.1% vs. gnomAD 0.1%). The second novel variant (p. Ser67Leu) were observed in 39 heterozygous carriers and one homozygous individual (QGP MAF 0.3% vs. gnomAD MAF 0.0004%). Functional characterization of the novel variant is warranted to confirm their pathogenicity. Detailed phenotypic data of the homozygous individuals carrying *LDLRAP1* variants are summarized in Supplementary Table 4.

We have identified one ClinVar reported pathogenic variant in *ABCG5* (p. Arg446*) associated with Sitosterolemia 1 present in 27 heterozygous carriers and no homozygous individual in QGP cohort (Tables 4 and 5). Furthermore, two ClinVar reported, and one novel putative pathogenic variant were identified in *ABCG8* including one homozygous individual carrying a known variant (p. Gly574Arg) associated with Sitosterolemia 2, explaining their higher LDL-C level of 6mmol/L (97th percentile) (Tables 4 and 5). Finally, we observed one ClinVar pathogenic and one novel putative pathogenic variant in LIPA, both in heterozygous state, associated with lysosomal acid lipase deficiency (Tables 4 and 5).

### Prevalence of known FH variants

We sought to determine the odds in the Qatari cohort of having a known pathogenic FH variant (*LDLR* variant) according to DLCN criteria. We found that 12% of the ‘definite or probable’ FH, 0.6% possible FH and 0.07% ‘unlikely’ FH individuals carried a known *LDLR* pathogenic variant. Further, the odds ratio (OR) of carrying a FH variant has been estimated to be 201 (95% CI: 55–736) for ‘definite or probable’ FH, and 7 (95% CI: 1-47) for 'possible' FH when compared to ‘unlikely’ FH (Supplementary Table 5).

### Assessing polygenic risk of FH

**Fig. 4:**
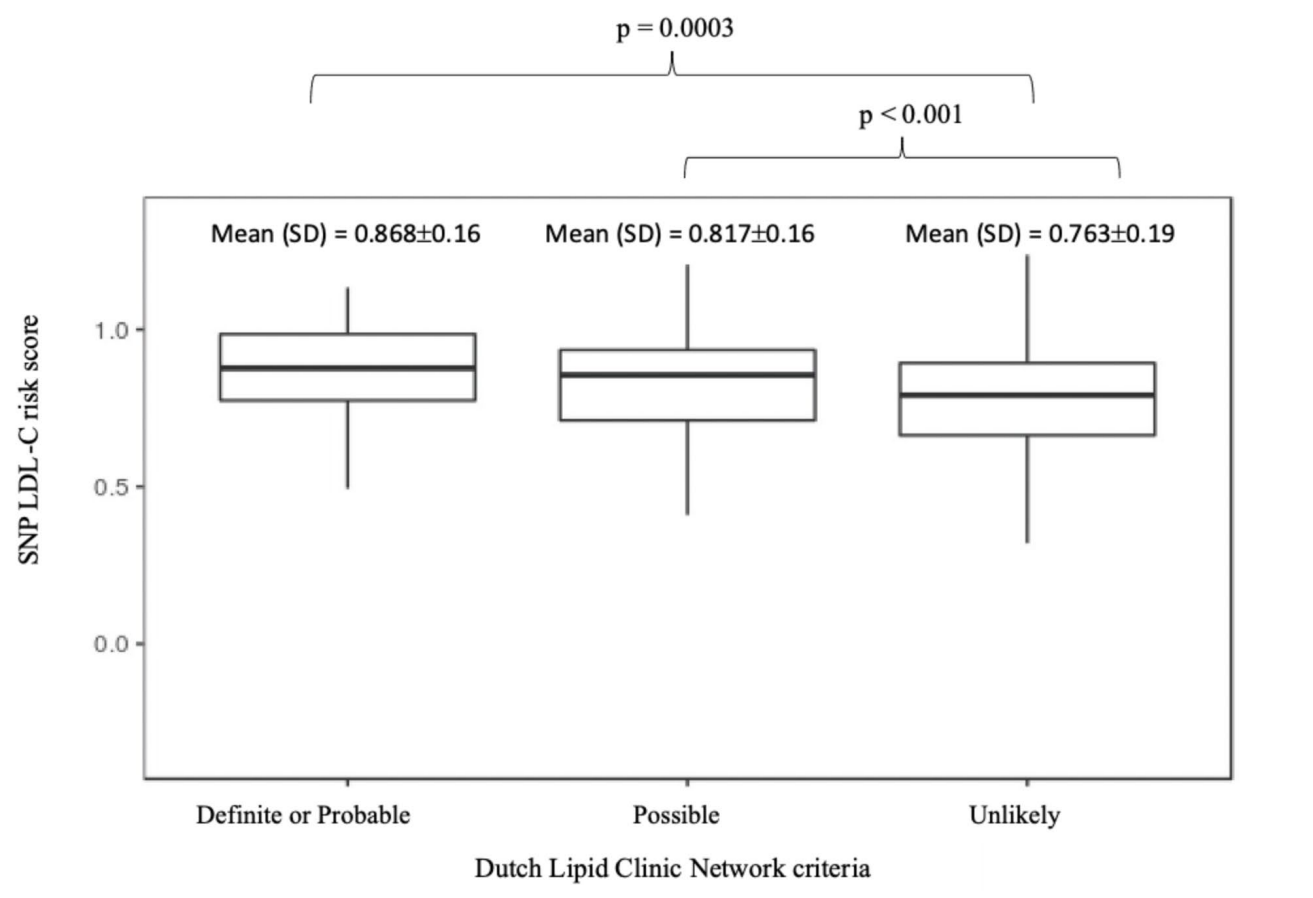
LDL-C SNP score for Dutch Lipid Clinic Network criteria. 12 SNP LDL-C SNP scores are represented as mean±SD. Compared to ‘unlikely’ FH group, both ‘definite or probable’ FH and ‘possible’ FH groups have significantly higher LDL-C scores (one-way ANOVA). However, there is no statistical difference observed between ‘definite or probable’ FH and ‘possible’ FH groups (p=0.17)

In addition to identifying single rare variants with large-effect, we investigated the contribution of 12 common variants to LDL-C levels in the Qatari cohort [14, 33–35, 46]. Based on the ‘unlikely’ FH individuals SNP LDL-C score distribution, we found 90% *LDLR* mutation-negative ‘definite or probable’ FH individuals had SNP scores above the bottom quartile (> 0.66) suggesting that high LDL-C in these individuals is likely to be due to polygenic contribution. Further, we found that 12 SNP LDL-C scores in ‘definite or probable’ FH individuals (0.87 ± 0.16 (mean ± SD)) and ‘possible’ FH individuals (0.82 ± 0.16) were significantly higher than in ‘unlikely’ FH (0.76 ± 0.19) (p-value < 0.01) (Fig. 4).

## Discussion

Familial hypercholesterolemia is the most common genetic cause of premature CAD [47] caused mainly by genetic variants in *LDLR*, *APOB* and *PCSK9* genes. Although the global prevalence is estimated to be between 1:250 to 1:500, knowledge of FH variants and its prevalence in the Middle East region has not been well established due to the lack of local or national registries [48]. By using DLCN criteria, we identified 0.1% [8] definite, 0.7% [41] probable, and 5% (334) possible FH individuals. This suggests a estimated prevalence of ‘definite or probable’ FH individuals in the QBB cohort of 1:125 (0.8%). The findings are comparable with those of Gulf FH registry study (Saudi Arabia, Oman, United Arab Emirates, Kuwait, and Bahrain) of 34,366 patients, which estimated a prevalence of FH (definite or probable) of 1:232 (0.43%) in the region. [47].

Studies indicate that 60–80% of those with a clinical diagnosis of ‘definite’ FH and 30% of ‘possible’ FH individuals have pathogenic variants in at least one of the three FH-causing genes [49]. However in our cohort, we observed the FH variants in 12% of the ‘definite or probable’ FH and 0.6% of possible FH. The mutation rates observed in our study were low compared to those observed in lipid clinic patients, such as 63–80% for definite FH individuals (DLCN criteria) [50–52]. A possible explanation may relate to the bias in referrals of patients with severe phenotypes to lipid clinics as compared to individuals with FH in the general population. Nevertheless, a community-based study, such as the Copenhagen general population study, reports mutation rates of 7.3% among those with ‘definite or probable’ FH and 1.2% among those with possible FH, in comparison to 12% and 0.6% for ‘definite or probable’ FH and possible FH, respectively, in our study.

Leveraging the WGS data from QGP, we identified ten ClinVar P/LP variants, 14 novel predicted pathogenic SNVs and a novel CNV in *PCSK9* among the 6,140 participants. The genetic architecture of the QGP participants relative to the world population reveals five major ancestries, which include general Arabs (QGP_GAR), peninsular Arabs (QGP_PAR), Arabs of Western Eurasia and Persia (QGP_WEP), South Asians (QGP_SAS), and Africans (QGP_AFR) [30]. The LOF variant (c.313 + 3 A > C) in the *LDLR* gene has been identified as the most common FH causing variant in Qatar and is found in six heterozygous individuals who all belong to the QGP_PAR subcluster (QGP_Penisular Arabs). Given the uniqueness of this variant to this relatively ancient and isolated genetic subgroup, it is likely that it has risen as a result of founder effect. This also implies that this variant may be unique to the Arab population, which is further supported by its absence from population databases. Despite the high degree of consanguinity [53], no homozygous individuals carrying known P/LP variants in the three candidate genes (*LDLR, APOB*, and *PCSK9*) were identified in the QGP cohort. This might be due to the severity of homozygous FH such that affected individuals do not survive past the second decade of life without treatment due to the very early risk of CAD. Also, the global prevalence of HoFH was estimated between 1:160,000 to 1:300,000 [9].

The cataloging of FH pathogenic variants and diagnostic classification of QBB participants allowed us to estimate the clinical penetrance of previously reported pathogenic variants in clinical databases. For the 28 variants annotated as disease-causing (DM) in the HGMD, for example, we observed complete penetrance for only four variants, incomplete penetrance (range: 6-67%) for six variants and remaining 18 DM variants had zero penetrance. Conversely, all three out of four ClinVar P/LP variants had high penetrance (≥50%). DM variants with zero penetrance might be attributable to: (i) the lack of sufficient carriers to estimate the actual estimated clinical penetrance or (ii) the possibility of false positives in the HGMD database [54].

A novel whole gene duplication of the *PCSK9* was observed in an individual with high LDL-C level (6.03 mmol/L). This is consistent with a previous report of two cases with an entire *PCSK9* duplication causing severe FH [55]. Structural mapping of the predicted pathogenic novel SNVs in *PCSK9* (p. Arg303His, p. Ala68Asp, p. Gly59Arg) and *LDLR* (p. Asp472Asn) suggest that they are positioned in functionally critical regions of the *PCSK9* and *LDLR* proteins, respectively.

In the QGP cohort, homozygous individuals carrying recessive FH variants were observed in *LDLRAP1* and *ABCG8* genes. Among the two *LDLRAP1* variants, the variant (p. Ser202Tyr) was among the first six mutations identified in the *LDLRAP1* gene by Garcia et al. (2001) in a Lebanon family, which was described as the ARH4 allele [56]. Two sisters from Lebanon, aged 7 and 17, carry this mutation with LDL-C levels of 10.1 mmol/L and 13.4 mmol/L, respectively. The siblings who carry the ARH4 allele also have a family history of CAD, and the father died at the age of 28 from myocardial infarction [56]. A total of three homozygous individuals carrying this variant have been reported in the QGP cohort. All three homozygous individuals were self-reported for hypercholesterolemia, two of them were undergoing treatment with cholesterol lowering medications and one with diet management. Although homozygous individuals carrying this variant found in population databases (GME, gnomAD) might suggest the variant has a low/incomplete penetrance, we have observed that three homozygous individuals carrying this variant have been diagnosed with hypercholesterolemia, and two of them have undergone heart revascularization surgery.

The homozygous individual carrying the second *LDLRAP1* variant (NP_056442.2: c.200 C > T; Ser67Leu) was a 36-year-old male who had been diagnosed with hypercholesterolemia at the age of 31 and had undergone heart revascularization surgery. The parents of this homozygous individual were reportedly first cousins. He has been treated with cholesterol-lowering medications. There is no other co-morbidity, such as obesity, hypertension, or diabetes mellitus, reported by the participant. Furthermore, no homozygous individuals carrying this variant have been reported in gnomAD or GME. Pathogenic prediction tools indicate that this variant may be deleterious and is in the mutational hotspot of the protein, more specifically, in exon 2 of the PTB/PID domain, which is necessary for the LDLRAP1 protein to bind to the NPXY motif present in the cytoplasmic tail of the LDL receptor.

We found one homozygous individual and 4 heterozygous carriers carry the known pathogenic *ABCG8* variant (p. Gly574Arg). The homozygous individual carrying the *ABCG8* variant (p. Gly574Arg) was a 47-year-old male who self-reported hypercholesterolemia and was treated with cholesterol-lowering medications and diet management. His parents were reported to be first cousins. A LDL-C level of 6 mmol/L was reported for this participant along with a total cholesterol level of 8 mmol/L, triglyceride level of 2.1 mmol/L, and HDL-C level of 1.03 mmol/L; however, his plant sterol level could not be determined because QBB does not have these data. While he has not had premature coronary artery disease, he has a family history of coronary artery disease and his father died of a heart attack. Other comorbid conditions include obesity with a BMI of 25.9, but no diabetes or hypertension was noted. This mutation was identified previously in a large Amish family in which a 13-year-old boy died of coronary atherosclerosis [57, 58]. Five of his twelve siblings developed tendon and tuberous xanthomas, as well as increased plasma plant sterols, particularly β -sitosterol.

While there are no published data regarding the prevalence of Sitosterolemia 2 [59], it appears to be more common in Caucasians [59, 60]. In contrast, Sitosterolemia 1 caused by *ABCG5* is more prevalent in Indians, Chinese, and Japanese [59]. Based on LOF variants identified in the ExAC database, the global prevalence of Sitosterolemia 2 is estimated to be at least 1 in 360,000 and 1 in 2.6 million for Sitosterolemia 1 [59]. The prevalence of Sitosterolemia 2 in QGP was 1:6140, which is high in comparison with the estimated global prevalence of 1:360,000.

The *LIPA* variant (p. Thr288Ile) found in one heterozygous carrier was associated with childhood onset Lysosomal Acid lipase Deficiency (LAL-D) (previously known as cholesteryl ester storage disease (CESD)). This variant was reported already in an Italian child in a homozygous state with the age of onset being 2 and showed the clinical characteristics of hepatosplenomegaly, dyslipidemia, and elevated transaminases [61].

Predicting the cause of clinical FH, whether monogenic or polygenic, can help clinicians to select the most effective and inexpensive lipid-lowering medications, representing the best example of the use of genetic information in precision medicine [13]. We investigated the 12 SNPs LDL-C raising scores, which the Bristol Genetics laboratories currently use in the UK for genetic screening of patients with a clinical diagnosis of FH [62]. We observed that 90% of mutation negative ‘definite or probable’ FH individuals had SNP scores within the top three quartiles of the unlikely FH individuals SNP score distribution, thus suggesting polygenic cause. This finding correlates with previous study in a European-Caucasian population, which concluded that 80% of mutation-negative clinically diagnosed FH patients have a polygenic inheritance as an explanation for their high cholesterol [13, 34]. Further, we observed that ‘definite or probable’ FH individuals, and ‘possible’ FH individuals had significantly higher LDL-C SNP scores than ‘unlikely’ FH individuals. Our results confirm the hypothesis that individuals at risk of hypercholesterolemia are highly expected to carry common LDL-C-raising alleles and might have polygenic inheritance. Further, we demonstrate that the 12-SNP LDL-C SNP score can be used to assess polygenic risk in Arab populations, although these SNPs are derived from Caucasians.

Despite the important findings of our study, there are some limitations. It should be noted that QBB phenotypic data lacks clinical features, such as tendon xanthomas or corneal arcularis, in the participants and the first-degree relatives, which are usually assigned higher scores in the DLCN criteria. However, the same limitations were also observed in other general population studies, such as the Copenhagen study, while using DLCN diagnostic criteria for FH diagnosis in 98,098 participants [1].

## Conclusion

Our study annotates a large-scale population biobank using DLCN diagnostic criteria for FH, and identifies known and putatively novel FH genetic variants present in the Qatari population. Knowledge of these variants and their further testing in ethnically Arab populations is important for clinical care and personalized medicine.

## Electronic Supplementary Material

Below is the link to the electronic supplementary material.

Supplementary Material 1

## Data Availability

Genotypic data of this study is accessed through a dedicated portal by QGP (Accession ID: QF-QGP-RES-PUB-003). According to the study participant’s informed consent, posting their phenotypic and genotypic data is not allowed in public databases. QBB/QGP data can be obtained through an established ISO-certified process by submitting a project request at https://www.qatarbiobank.org.qa/research/how-apply, which is subject to approval by the QBB IRB committee.

## List of abbreviations

*ABCG5*: ATP-Binding Cassette Sub-Family G Member 5
*ABCG8*: ATP-Binding Cassette Sub-Family G Member 8
ACMG: American College of Medical Genetics
*APOB*: Apolipoprotein B
CAD: Coronary artery diseases
FH: Familial Hypercholesterolemia
FHSC: EAS Familial Hypercholesterolaemia Studies Collaboration
GLGC: Global Lipid Genetics Consortium
HDL: High-Density Lipoproteins
HGMD: Human Genome Mutation Database
HoFH: Homozygous FH Cases
*LDLR*: Low-Density Lipoprotein Receptor
*LDLRAP1*: Low-Density Lipoprotein Receptor Adaptor Protein 1
*LIPA*: Lipase A
LOF: Loss-Of-Function
MI: Myocardial Infarction
NGS: Next-Generation Sequencing
OR: Odds ratio
*PCSK9*: Proprotein Convertase Subtilisin/Kexin type 9
QBB: Qatar Biobank
QGP: Qatar Genome Program
TSH: Thyroid stimulating Hormone
WGS: Whole-Genome Sequencing

## References

[1] Benn M, Watts GF, Tybjaerg-Hansen A, Nordestgaard BG (2016). Mutations causative of familial hypercholesterolaemia: screening of 98 098 individuals from the Copenhagen General Population Study estimated a prevalence of 1 in 217. Eur Heart J.

[2] Hopkins PN, Toth PP, Ballantyne CM, Rader DJ, National Lipid Association Expert Panel on Familial H (2011). Familial hypercholesterolemias: prevalence, genetics, diagnosis and screening recommendations from the National Lipid Association Expert Panel on Familial Hypercholesterolemia. J Clin Lipidol.

[3] Marks D, Thorogood M, Neil HA, Humphries SE (2003). A review on the diagnosis, natural history, and treatment of familial hypercholesterolaemia. Atherosclerosis.

[4] Sjouke B, Kusters DM, Kindt I, Besseling J, Defesche JC, Sijbrands EJ (2015). Homozygous autosomal dominant hypercholesterolaemia in the Netherlands: prevalence, genotype-phenotype relationship, and clinical outcome. Eur Heart J.

[5] Gidding SS, Champagne MA, de Ferranti SD, Defesche J, Ito MK, Knowles JW (2015). The Agenda for Familial Hypercholesterolemia: A Scientific Statement From the American Heart Association. Circulation.

[6] Goldberg AC, Hopkins PN, Toth PP, Ballantyne CM, Rader DJ, Robinson JG (2011). Familial hypercholesterolemia: screening, diagnosis and management of pediatric and adult patients: clinical guidance from the National Lipid Association Expert Panel on Familial Hypercholesterolemia. J Clin Lipidol.

[7] Singh S, Bittner V (2015). Familial hypercholesterolemia–epidemiology, diagnosis, and screening. Curr Atheroscler Rep.

[8] Austin MA, Hutter CM, Zimmern RL, Humphries SE (2004). Genetic causes of monogenic heterozygous familial hypercholesterolemia: a HuGE prevalence review. Am J Epidemiol.

[9] Nordestgaard BG, Chapman MJ, Humphries SE, Ginsberg HN, Masana L, Descamps OS (2013). Familial hypercholesterolaemia is underdiagnosed and undertreated in the general population: guidance for clinicians to prevent coronary heart disease: consensus statement of the European Atherosclerosis Society. Eur Heart J.

[10] Risk of fatal coronary heart disease in familial hypercholesterolaemia (1991). Sci Steer Comm behalf Simon Broome Register Group BMJ.

[11] Williams RR, Hunt SC, Schumacher MC, Hegele RA, Leppert MF, Ludwig EH (1993). Diagnosing heterozygous familial hypercholesterolemia using new practical criteria validated by molecular genetics. Am J Cardiol.

[12] Goldstein JL, Hobbs HH, Brown MS, Valle DL, Antonarakis S, Ballabio A, Beaudet AL, Mitchell GA (2019). Familial Hypercholesterolemia. The Online Metabolic and Molecular Bases of Inherited Disease.

[13] Futema M, Bourbon M, Williams M, Humphries SE (2018). Clinical utility of the polygenic LDL-C SNP score in familial hypercholesterolemia. Atherosclerosis.

[14] Talmud PJ, Shah S, Whittall R, Futema M, Howard P, Cooper JA (2013). Use of low-density lipoprotein cholesterol gene score to distinguish patients with polygenic and monogenic familial hypercholesterolaemia: a case-control study. Lancet.

[15] Fahed AC, Safa RM, Haddad FF, Bitar FF, Andary RR, Arabi MT (2011). Homozygous familial hypercholesterolemia in Lebanon: a genotype/phenotype correlation. Mol Genet Metab.

[16] Khachadurian AK, Uthman SM (1973). Experiences with the homozygous cases of familial hypercholesterolemia. A report of 52 patients. Nutr Metab.

[17] Fellin R, Arca M, Zuliani G, Calandra S, Bertolini S (2015). The history of Autosomal Recessive Hypercholesterolemia (ARH). From clinical observations to gene identification. Gene.

[18] Soutar AK, Naoumova RP, Traub LM (2003). Genetics, clinical phenotype, and molecular cell biology of autosomal recessive hypercholesterolemia. Arterioscler Thromb Vasc Biol.

[19] D’Erasmo L, Di Costanzo A, Arca M (2020). Autosomal recessive hypercholesterolemia: update for 2020. Curr Opin Lipidol.

[20] Pisciotta L, Priore Oliva C, Pes GM, Di Scala L, Bellocchio A, Fresa R (2006). Autosomal recessive hypercholesterolemia (ARH) and homozygous familial hypercholesterolemia (FH): a phenotypic comparison. Atherosclerosis.

[21] Collaboration EASFHS, Vallejo-Vaz AJ, De Marco M, Stevens CAT, Akram A, Freiberger T (2018). Overview of the current status of familial hypercholesterolaemia care in over 60 countries - The EAS Familial Hypercholesterolaemia Studies Collaboration (FHSC). Atherosclerosis.

[22] Alhababi D, Zayed H (2018). Spectrum of mutations of familial hypercholesterolemia in the 22 Arab countries. Atherosclerosis.

[23] Al Thani A, Fthenou E, Paparrodopoulos S, Al Marri A, Shi Z, Qafoud F (2019). Qatar Biobank Cohort Study: Study Design and First Results. Am J Epidemiol.

[24] Al Kuwari H, Al Thani A, Al Marri A, Al Kaabi A, Abderrahim H, Afifi N (2015). The Qatar Biobank: background and methods. BMC Public Health.

[25] Haralambos K, Whatley SD, Edwards R, Gingell R, Townsend D, Ashfield-Watt P (2015). Clinical experience of scoring criteria for Familial Hypercholesterolaemia (FH) genetic testing in Wales. Atherosclerosis.

[26] Jones PH, Davidson MH, Stein EA, Bays HE, McKenney JM, Miller E (2003). Comparison of the efficacy and safety of rosuvastatin versus atorvastatin, simvastatin, and pravastatin across doses (STELLAR* Trial). Am J Cardiol.

[27] Alberti KG, Eckel RH, Grundy SM, Zimmet PZ, Cleeman JI, Donato KA, et al. Harmonizing the metabolic syndrome: a joint interim statement of the International Diabetes Federation Task Force on Epidemiology and Prevention; National Heart, Lung, and Blood Institute; American Heart Association; World Heart Federation; International Atherosclerosis Society; and International Association for the Study of Obesity. Circulation. 2009;120(16):1640-5. 10.1161/CIRCULATIONAHA.109.192644 19805654

[28] Mbarek H, Gandhi GD, Selvaraj S, Al-Muftah W, Badji R, Al-Sarraj Y, et al. Qatar Genome: Insights on Genomics from the Middle East. medRxiv. 2021:2021.09.19.21263548. 10.1002/humu.24336 35112413

[29] Thareja G, Al-Sarraj Y, Belkadi A, Almotawa M, Qatar Genome Program Research C, Suhre K (2021). Whole genome sequencing in the Middle Eastern Qatari population identifies genetic associations with 45 clinically relevant traits. Nat Commun.

[30] Razali RM, Rodriguez-Flores J, Ghorbani M, Naeem H, Aamer W, Aliyev E (2021). Thousands of Qatari genomes inform human migration history and improve imputation of Arab haplotypes. Nat Commun.

[31] Abul-Husn NS, Manickam K, Jones LK, Wright EA, Hartzel DN, Gonzaga-Jauregui C, et al. Genetic identification of familial hypercholesterolemia within a single U.S. health care system. Science. 2016;354(6319). 10.1126/science.aaf7000 28008010

[32] Li Q, Wang K, InterVar (2017). Clinical Interpretation of Genetic Variants by the 2015 ACMG-AMP Guidelines. Am J Hum Genet.

[33] Bennet AM, Di Angelantonio E, Ye Z, Wensley F, Dahlin A, Ahlbom A (2007). Association of apolipoprotein E genotypes with lipid levels and coronary risk. JAMA.

[34] Futema M, Shah S, Cooper JA, Li K, Whittall RA, Sharifi M (2015). Refinement of variant selection for the LDL cholesterol genetic risk score in the diagnosis of the polygenic form of clinical familial hypercholesterolemia and replication in samples from 6 countries. Clin Chem.

[35] Teslovich TM, Musunuru K, Smith AV, Edmondson AC, Stylianou IM, Koseki M (2010). Biological, clinical and population relevance of 95 loci for blood lipids. Nature.

[36] Kerr M, Pears R, Miedzybrodzka Z, Haralambos K, Cather M, Watson M (2017). Cost effectiveness of cascade testing for familial hypercholesterolaemia, based on data from familial hypercholesterolaemia services in the UK. Eur Heart J.

[37] Elfatih A, Mifsud B, Syed N, Badii R, Mbarek H, Abbaszadeh F, et al. Actionable genomic variants in 6045 participants from the Qatar Genome Program. Hum Mutat. 2021. 10.1002/humu.24278 34428338

[38] Anderson ED, Molloy SS, Jean F, Fei H, Shimamura S, Thomas G (2002). The ordered and compartment-specfific autoproteolytic removal of the furin intramolecular chaperone is required for enzyme activation. J Biol Chem.

[39] Baker D, Shiau AK, Agard DA (1993). The role of pro regions in protein folding. Curr Opin Cell Biol.

[40] Kwon HJ, Lagace TA, McNutt MC, Horton JD, Deisenhofer J (2008). Molecular basis for LDL receptor recognition by PCSK9. Proc Natl Acad Sci U S A.

[41] Seidah NG, Poirier S, Denis M, Parker R, Miao B, Mapelli C (2012). Annexin A2 is a natural extrahepatic inhibitor of the PCSK9-induced LDL receptor degradation. PLoS ONE.

[42] Ly K, Saavedra YG, Canuel M, Routhier S, Desjardins R, Hamelin J (2014). Annexin A2 reduces PCSK9 protein levels via a translational mechanism and interacts with the M1 and M2 domains of PCSK9. J Biol Chem.

[43] Leren TP (2014). Sorting an LDL receptor with bound PCSK9 to intracellular degradation. Atherosclerosis.

[44] Rudenko G, Henry L, Henderson K, Ichtchenko K, Brown MS, Goldstein JL (2002). Structure of the LDL receptor extracellular domain at endosomal pH. Science.

[45] Lo Surdo P, Bottomley MJ, Calzetta A, Settembre EC, Cirillo A, Pandit S (2011). Mechanistic implications for LDL receptor degradation from the PCSK9/LDLR structure at neutral pH. EMBO Rep.

[46] Olmastroni E, Gazzotti M, Arca M, Averna M, Pirillo A, Catapano AL (2022). Twelve Variants Polygenic Score for Low-Density Lipoprotein Cholesterol Distribution in a Large Cohort of Patients With Clinically Diagnosed Familial Hypercholesterolemia With or Without Causative Mutations. J Am Heart Assoc.

[47] Al-Rasadi K, Alhabib KF, Al-Allaf F, Al-Waili K, Al-Zakwani I, AlSarraf A (2020). The Gulf Familial Hypercholesterolemia Registry (Gulf FH): Design, Rationale and Preliminary Results. Curr Vasc Pharmacol.

[48] Bamimore MA, Zaid A, Banerjee Y, Al-Sarraf A, Abifadel M, Seidah NG (2015). Familial hypercholesterolemia mutations in the Middle Eastern and North African region: a need for a national registry. J Clin Lipidol.

[49] Taylor A, Wang D, Patel K, Whittall R, Wood G, Farrer M (2010). Mutation detection rate and spectrum in familial hypercholesterolaemia patients in the UK pilot cascade project. Clin Genet.

[50] Civeira F, Ros E, Jarauta E, Plana N, Zambon D, Puzo J (2008). Comparison of genetic versus clinical diagnosis in familial hypercholesterolemia. Am J Cardiol.

[51] Damgaard D, Larsen ML, Nissen PH, Jensen JM, Jensen HK, Soerensen VR (2005). The relationship of molecular genetic to clinical diagnosis of familial hypercholesterolemia in a Danish population. Atherosclerosis.

[52] Heath KE, Humphries SE, Middleton-Price H, Boxer M (2001). A molecular genetic service for diagnosing individuals with familial hypercholesterolaemia (FH) in the United Kingdom. Eur J Hum Genet.

[53] Bener A, Alali KA (2006). Consanguineous marriage in a newly developed country: the Qatari population. J Biosoc Sci.

[54] Xue Y, Chen Y, Ayub Q, Huang N, Ball EV, Mort M (2012). Deleterious- and disease-allele prevalence in healthy individuals: insights from current predictions, mutation databases, and population-scale resequencing. Am J Hum Genet.

[55] Iacocca MA, Wang J, Sarkar S, Dron JS, Lagace T, McIntyre AD (2018). Whole-Gene Duplication of PCSK9 as a Novel Genetic Mechanism for Severe Familial Hypercholesterolemia. Can J Cardiol.

[56] Garcia CK, Wilund K, Arca M, Zuliani G, Fellin R, Maioli M (2001). Autosomal recessive hypercholesterolemia caused by mutations in a putative LDL receptor adaptor protein. Science.

[57] Kwiterovich PO, Bachorik PS, Smith HH, McKusick VA, Connor WE, Teng B (1981). Hyperapobetalipoproteinaemia in two families with xanthomas and phytosterolaemia. Lancet.

[58] Horenstein RB, Mitchell BD, Post WS, Lutjohann D, von Bergmann K, Ryan KA (2013). The ABCG8 G574R variant, serum plant sterol levels, and cardiovascular disease risk in the Old Order Amish. Arterioscler Thromb Vasc Biol.

[59] Hooper AJ, Bell DA, Hegele RA, Burnett JR. Clinical utility gene card for: Sitosterolaemia. Eur J Hum Genet. 2017;25(4). 10.1038/ejhg.2016.187 PMC5386411 28029149

[60] Lu K, Lee MH, Hazard S, Brooks-Wilson A, Hidaka H, Kojima H (2001). Two genes that map to the STSL locus cause sitosterolemia: genomic structure and spectrum of mutations involving sterolin-1 and sterolin-2, encoded by ABCG5 and ABCG8, respectively. Am J Hum Genet.

[61] Pisciotta L, Tozzi G, Travaglini L, Taurisano R, Lucchi T, Indolfi G (2017). Molecular and clinical characterization of a series of patients with childhood-onset lysosomal acid lipase deficiency. Retrospective investigations, follow-up and detection of two novel LIPA pathogenic variants. Atherosclerosis.

[62] Leal LG, Hoggart C, Jarvelin MR, Herzig KH, Sternberg MJE, David A (2020). A polygenic biomarker to identify patients with severe hypercholesterolemia of polygenic origin. Mol Genet Genomic Med.

