# Supplementary material for: Assessing the genetic burden of familial hypercholesterolemia in a large middle eastern biobank
Source: J Transl Med. 2022 Nov 3;20:502. doi: 10.1186/s12967-022-03697-w (PMC9635206; doi:10.1186/s12967-022-03697-w)
Supplement: Supplementary file 1 — Supplementary Material 1 [file 12967_2022_3697_MOESM1_ESM.docx]

**Supplemental Table 1. Cholesterol lowering medications and the corresponding correction factors used in this study.**

| **Statin/dose (mg)** | **Correction factor** |
| --- | --- |
| **Ezetimibe** | |
| 10 | 1.2 |
| Dosage not available | 1.2 |
| **Pravastatin** | |
| 10 | 1.2 |
| 20 | 1.3 |
| 40 | 1.5 |
| Dosage not available | 1.3 |
| **Pravastatin + Ezetimibe** | |
| 10 + 10 | 1.5 |
| 20 + 10 | 1.6 |
| 40 + 10 | 1.7 |
| **Simvastatin** | |
| 10 | 1.4 |
| 20 | 1.6 |
| 40 | 1.7 |
| 80 | 1.9 |
| Dosage not available | 1.65 |
| **Simvastatin + Ezetemibe** | |
| 10 + 10 | 1.9 |
| 20 + 10 | 2 |
| 40 + 10 | 2.3 |
| 80 + 10 | 2.4 |
| **Atorvastatin** | |
| 10 | 1.6 |
| 20 | 1.8 |
| 40 | 2 |
| 80 | 2.2 |
| Dosage not available | 1.9 |
| **Atorvastatin + Ezetimibe** | |
| 10 + 10 | 2 |
| 20 + 10 | 2.2 |
| 40 + 10 | 2.2 |
| 80 + 10 | 2.5 |
| **Rosuvastatin** | |
| 5 | 1.8 |
| 10 | 1.9 |
| 20 | 2.1 |
| 40 | 2.4 |
| Dosage not available | 2 |
| **Rosuvastatin + Ezetimibe** | |
| 10 + 10 | 2.5 |
| 20 + 10 | 2.7 |
| 40 + 10 | 3.3 |
| Dosage not available | 2.7 |
| **Others** | 1.43 |

A previously described correction factor was used for each class of statins (Haralambos et al., 2015). In cases where the drug information did not include the dosage information, the median of those particular statin groups was used as the correction factor. ‘Others’ includes the QBB participants treated with an unspecified cholesterol-lowering medication. For the QBB participants in the ‘other’ category, a correction factor of 1.43 was used, corresponding to an estimated 30% reduction in LDL-cholesterol achieved with standard cholesterol lowering medication.

**Supplemental Table 2. SNPs and haplotypes used to calculate the LDL-C SNP risk score.**

| **Chr** | **dbSNP ID** | **Gene** | **Minor allele** | **Common allele** | **GLGC weight for score calculation** |
| --- | --- | --- | --- | --- | --- |
| 1 | rs2479409 | *PCSK9* | G* | A | 0·052 |
| 1 | rs629301 | *CELSR2* | G | T* | 0·15 |
| 2 | rs1367117 | *APOB* | A* | G | 0·10 |
| 2 | rs4299376 | *ABCG8* | G* | T | 0·071 |
| 6 | rs1564348 | *SLC22A1* | C | T* | 0·014 |
| 6 | rs3757354 | *MYLIP* | T | C* | 0·037 |
| 11 | rs11220462 | *ST3GAL4* | A* | G | 0·050 |
| 14 | rs8017377 | *NYNRIN* | A* | G | 0·029 |
| 19 | rs6511720 | *LDLR* | T | G* | 0·18 |
| 19 | rs429358 | *APOE†* | C | T | ·· |
| 19 | rs7412 | *APOE†* | T | C | ·· |
| 19 | ε2ε2 | *APOE* | ·· | ·· | −0·9 |
| 19 | ε2ε3 | *APOE* | ·· | ·· | −0·4 |
| 19 | ε2ε4 | *APOE* | ·· | ·· | 0·2 |
| 19 | ε3ε3 | *APOE* | ·· | ·· | 0 |
| 19 | ε3ε4 | *APOE* | ·· | ·· | 0·1 |
| 19 | ε4ε4 | *APOE* | ·· | ·· | 0·2 |
|  |  |  |  |  |  |

*****Risk alleles (LDL-C-raising). GLGC (Global Lipid Genetics Consortium) weights obtained from Kerr *et al* 2017.

†*APOE* weights were based on haplotypic effects described by Bennet *et al* 2007*.*

**Supplementary Table 3**: **HGMD Disease-causing (DM) mutations associated with Familial Hypercholesterolemia (FH) in the Qatar Genome Program study.**

| **Gene** | **dbSNP** | **cDNA change** | **Amino-acid change** | **QGP AC** | **QGP subcluster** | **QGP estimated clinical penetrance** | **ClinVar significance** |  |
| --- | --- | --- | --- | --- | --- | --- | --- | --- |
| *ABCG5* | rs199689137 | c.1336C>T | p.Arg446* | 27 | QGP_ADM (1), QGP_GAR (24), QGP_PAR (1), QGP_WEP (1) | NA | P |  |
| *ABCG5* |  | c.211G>A | p.Asp71Asn | 1 | QGP_AFR (1) | NA | . |  |
| *ABCG8* | rs137852991 | c.1234C>T | p.Arg412* | 1 | QGP_ADM (1) | NA | P |  |
| *ABCG8* | rs769576789 | c.1715T>C | p.Leu572Pro | 1 | QGP_WEP (1) | NA | VUS |  |
| *ABCG8* | rs137852988 | c.1720G>A | p.Gly574Arg | 6 | QGP_WEP (6) | 100% (1)* | P/LP |  |
| *APOB* | rs530171166 | c.1199G>A | p. Arg400His | 15 | QGP _ADM (2), QGP_SAS (2), QGP_WEP (11) | 7% (1/15) | LB |  |
| *APOB* | rs13306190 | c.1298C>T | p. Ala433Val | 1 | QGP_WEP | 0% (0/1) | LB |  |
| *LDLR* | rs776421777 | c.91G>A | p. Glu31Lys | 1 | QGP_ADM | 0% (0/1) | CIP |  |
| *LDLR* | rs376207800 | c.185C>T | p. Thr62Met | 8 | QGP_ADM (1), QGP_GAR (4), QGP_WEP (3) | 14% (1/7) | VUS |  |
| *LDLR* | rs771019366 | c.269A>G | p. Asp90Gly | 3 | QGP_ADM (1), QGP_WEP (2) | 67% (2/3) | P/LP |  |
| *LDLR* | rs201102461 | c.344G>A | p. Arg115His | 1 | QGP_GAR | 0% (0/1) | CIP |  |
| *LDLR* | rs10417394 | c.498C>T | p. Ala166Ala | 9 | QGP_ADM (6), QGP_AFR (1), QGP_GAR (2) | 0% (0/9) | CIP |  |
| *LDLR* | rs72658858 | c.940G>A | p. Gly314Arg | 17 | QGP_ADM (9, QGP_GAR (8) | 0% (0/17) | VUS |  |
| *LDLR* | rs747507019 | c.979C>T | p. His327Tyr | 1 | QGP_WEP | 100% (1/1) | CIP |  |
| *LDLR* | rs139361635 | c.1024G>A | p. Asp342Asn | 2 | QGP_ADM | 0% (0/2) | CIP |  |
| *LDLR* | rs752951310 | c.1145G>T | p. Gly382Val | 1 | QGP_AFR | 100% (1/1) | LP |  |
| *LDLR* | rs879254809 | c.1154T>G | p. Leu385Arg | 1 | QGP_AFR | 0% (0/1) | LP |  |
| *LDLR* | rs730882100 | c.1294C>G | p. Leu432Val | 3 | QGP_ADM (1), QGP_GAR (1), QGP_WEP (1) | 0% (0/3) | CIP |  |
| *LDLR* | rs745343524 | c.1301C>T | p. Thr434Met | 1 | QGP_GAR | 0% (0/1) | CIP |  |
| *LDLR* | rs780316072 | c.1429G>A | p. Asp477Asn | 2 | QGP_ADM | 0% (0/2) | VUS |  |
| *LDLR* | rs730882103 | c.1510A>G | p. Lys504Glu | 5 | QGP_WEP | 20% (1/5) | CIP |  |
| *LDLR* | rs879254974 | c.1658A>G | p. Tyr553Cys | 1 | QGP_AFR | 0% (0/1) | VUS |  |
| *LDLR* | rs758194385 | c.1691A>G | p. Asn564Ser | 1 | QGP_GAR | 100% (1/1) | LP |  |
| *LDLR* | rs763147599 | c.1774G>A | p. Gly592Arg | 2 | QGP_WEP | 50% (1/2) | P/LP |  |
| *LDLR* | rs72658865 | c.1816G>A | p. Ala606Thr | 1 | QGP_ADM | 0% (0/1) | CIP |  |
| *LDLR* | rs143872778 | c.1836C>T | p. Ala612Ala | 1 | QGP_ADM | 0% (0/1) | CIP |  |
| *LDLR* | rs368838866 | c.2101G>A | p. Gly701Ser | 2 | QGP_ADM (1), QGP_WEP (1) | 0% (0/2) | CIP |  |
| *LDLR* | rs750518671 | c.2389G>A | p. Ala797Thr | 1 | QGP_GAR | 100% (1/1) | CIP |  |
| *LDLR* | rs5928 | c.2503G>A | p. Gly835Ser | 4 | QGP_ADM (2), QGP_WEP (2) | 0% (0/4) | CIP |  |
| *LDLRAP1* | rs121908326 | c.605C>A | p.Ser202Tyr | 138 | QGP_ADM (34), QGP_AFR (3), QGP_GAR (81), QGP_PAR (8), QGP_WEP (12) | 0% (0/3)* | CIP |  |
| *LIPA* |  | c.863C>T | p.Thr288Ile | 1 | QGP_SAS (1) | NA | LP |  |
| *LIPA* | rs2228159 | c.683T>C | p.Phe228Ser | 1 | QGP_ADM (1) | NA | CIP |  |
| *PCSK9* | rs569379713 | c.212C>T | p. Pro71Leu | 4 | QGP_ADM (1), QGP_WEP (3) | 0% (0/4) | VUS |  |
| *PCSK9* | rs185392267 | c.286C>T | p. Arg96Cys | 2 | QGP_ADM (2), QGP_WEP (2) | 0% (0/2) | CIP |  |
| *PCSK9* | rs148562777 | c.1069C>T | p. Arg357Cys | 5 | QGP_GAR (4), QGP_WEP (1) | 0% (0/5) | VUS |  |
| *PCSK9* | rs141502002 | c.1405C>T | p. Arg469Trp | 34 | QGP_ADM (7), QGP_AFR (4), QGP_GAR (11), QGP_WEP (12) | 6% (2/34) | CIP |  |
|  |  |  |  |  |  |  |  |  |

* Estimated clinical penetrance was calculated for the homozygous individuals carrying the recessive variant. NA indicates no homozygous individuals available to calculate the penetrance for these recessive variants.

*ABCG5 -* ATP Binding Cassette Subfamily G Member 5, *ABCG8* - ATP Binding Cassette Subfamily G Member 8, *APOB* - apolipoprotein B, *LDLR* - Low-density lipoprotein receptor, *LDLRAP1 -* Low-Density Lipoprotein Receptor Adaptor Protein 1, *LIPA* - Lipase A, Lysosomal Acid Type, and *PCSK9* - proprotein convertase subtilisin/kexin type 9.

ClinVar significance: LB - likely benign; VUS - variants of uncertain significance; CIP - conflicting interpretations of pathogenicity; LP - likely pathogenic and P - pathogenic.

**Supplemental Table 4. Qatar Biobank phenotypic data for homozygous individuals carrying *LDLRAP1* and *ABCG8* variants.**

| **Phenotypic data** | ***LDLRAP1*** | | | | ***ABCG8*** |
| --- | --- | --- | --- | --- | --- |
|  | **NP_056442.2: p. Ser202Tyr** | | | **NP_056442.2:**  **p. Ser67Leu** | **NP_071882: p.Gly574Arg** |
|  | **Homozygous individual 1** | **Homozygous individual 2** | **Homozygous individual 3** | **Homozygous individual 1** | **Homozygous individual 1** |
| Age | 69 | 48 | 60 | 36 | 47 |
| Gender | Male | Male | Male | Male | Male |
| Parental consanguinity | First cousin | Double first cousin | NA | First cousin | First Cousin |
| Self-reported hypercholesterolemia | Yes | Yes | Yes | Yes | Yes |
| Age of diagnosis | 30 | NA | 55 | 31 | NA |
| Treatment for Hypercholesterolemia | Cholesterol lowering medication and diet | Diet only | No | Cholesterol lowering medication | Cholesterol lowering medication and diet |
| History of premature coronary artery diseases | None | None | Heart attack | None | None |
| History of surgery | Heart revascularization (bypass, angioplasty, coronary atherectomy) | None | Heart revascularization (bypass, angioplasty, coronary atherectomy) | Heart revascularization (bypass, angioplasty, coronary atherectomy) | None |
| Parental history of CAD | Mother suffers/suffered from heart diseases | Mother suffers/suffered from heart diseases | None | None | Father died of heart attack |
| Body mass index | 37.4 | 23.7 | 34.1 | 22.2 | 25.9 |
| Obesity | Yes | No | Yes | No | Yes |
| Diabetes mellitus | Yes | No | No | No | No |
| Hypertension | Yes | No | No | No | No |
| Metabolic syndrome | Yes | No | Yes | No | No |

**Supplementary Table 5: The odds ratio of having a low-density lipoprotein receptor gene variant in the QBB participants**

**classified according to Dutch Lipid Clinic Network criteria.**

| Dutch Lipid Clinic Network criteria | No. of QBB participants  (n=6140) | *LDLR* carriers (n=11) | OR (95% CI) | *P*-value |
| --- | --- | --- | --- | --- |
| Definite or Probable FH | 49 | 6 | 201 (55-736) | < 0.0001 |
| Possible FH | 334 | 2 | 7 (1-47) | 0.01 |
| Unlikely FH | 5757 | 4 | 1 | . |

OR - Odds Ratio; CI – Confidence Interval.


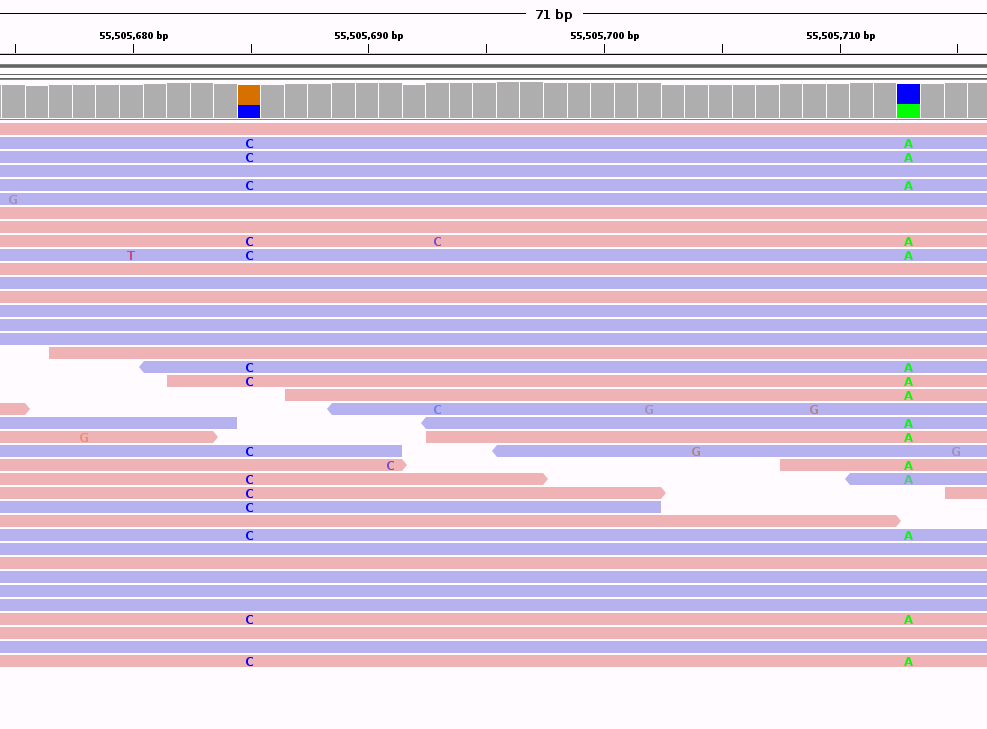


**Supplementary Figure 1. Visualization of two novel *PCSK9* mutations (c.175G>C and c.203C>A) by Integrative Genomics Viewer (IGV).**
